# Case report: Bilateral posterior ischemic optic neuropathy in a patient with atrial fibrillation and multifocal embolic stroke

**DOI:** 10.3389/fneur.2022.988825

**Published:** 2022-12-02

**Authors:** Jin-Ju Kang, Eun-Su Lee, Haeng-Jin Lee, Seungbae Hwang, Myung-Ja Chung, Sun-Young Oh

**Affiliations:** ^1^Department of Neurology, Jeonbuk National University Hospital and School of Medicine, Jeonju, South Korea; ^2^Research Institute of Clinical Medicine of Jeonbuk National University-Jeonbuk National University Hospital, Jeonju, South Korea; ^3^Department of Ophthalmology, Jeonbuk National University Hospital and School of Medicine, Jeonju, South Korea; ^4^Department of Radiology, Jeonbuk National University Hospital and School of Medicine, Jeonju, South Korea; ^5^Department of Pathology, Jeonbuk National University Hospital School of Medicine, Jeonju, South Korea

**Keywords:** ischemic optic neuropathy, optic nerve, posterior ischemic optic neuropathy (PION), atrial fibrillation (AF), embolic stroke

## Abstract

An 80-year-old female with a history of diabetes mellitus (DM) and hypertension presented with sudden onset of sequential bilateral visual loss. The best visual acuity was light perception in the right eye and finger counting in the left eye, however, bilateral fundus did not reveal optic disc edema. Diffusion-weighted magnetic resonance imaging (MRI) of the brain revealed acute embolic stroke and diffusion restriction in the posterior portion of both optic nerves. The 24-h Holter monitor showed persistent atrial fibrillation (AF) with rapid ventricular response. The presence of painless and severe visual loss at onset unaccompanied by optic disc edema in the patient with newly detected uncontrolled AF and multiple embolic infarctions favored a diagnosis of non-arteritic posterior ischemic optic neuropathy (PION). The current case contributes to better understanding of PION pathophysiology and associated risk factors, indicating a possible relationship between non-arteritic PION and uncontrolled AF and embolic cerebral infarction.

## Introduction

Posterior ischemic optic neuropathy (PION) is a rare optic neuropathy of vascular origin involving the retrobulbar optic nerve and unaccompanied by optic disc edema in contrast to anterior ION (AION), which involves the optic nerve head and presents with disc edema. In a previous study, 1,400 ION patients were analyzed and the relative frequency of AION and PION was reportedly 96 and 4%, respectively (1). PION is usually a diagnosis of exclusion when other causes of retrobulbar optic neuropathy such as inflammation, toxicity, and compression are eliminated. PION can be classified into three main categories based on etiology: perioperative, arteritic, and non-arteritic (2). Perioperative PION more likely occurs postoperatively and may present with bilateral optic nerve damage at a younger age with worse initial and final visual outcomes (3). These patients have undergone surgery usually associated with a lengthy procedure, increased blood loss, and low blood pressure such as spine and cardiac surgery. Arteritic PION is mostly due to giant cell arteritis and patients are older with more severe visual loss and poor visual recovery. Non-arteritic PION, similar to non-arteritic AION, is a multifactorial disease with various systemic vascular or local risk factors predisposing an optic nerve to develop PION. The patients tend to have a significantly higher prevalence of diabetes mellitus (DM), arterial hypotension or hypertension, atherosclerosis, and cardiovascular disorders (4). Although this association does not necessarily indicate a cause-and-effect relationship with non-arteritic PION, they may constitute risk factors for the development of PION (4). Non-arteritic PION is a hypotensive disorder (e.g., nocturnal arterial hypotension) in the majority of cases and rarely a thromboembolic disorder. Conversely, ischemic cerebral stroke is usually a thromboembolic disorder. In several studies, the incidence of atrial fibrillation (AF) was reportedly higher in the retinal vascular ischemia group than in the control group, however, studies are lacking in which AF in patients with PION was investigated (5–7).

Typically, PION presents with monocular visual loss, especially the non-arteritic form. Simultaneous bilateral visual loss is usually encountered in the perioperative type of PION where perfusion abnormalities to the eyes occur, and the arteritic form may cause sequential progressive bilateral visual loss. Herein, we describe an elderly female patient who presented with sequential bilateral visual loss accompanied by multiple embolic cerebral infarcts and uncontrolled severe AF, diagnosed as non-arteritic PION, which has not been previously reported in the literature. This case contributes to better understanding of PION pathophysiology and the associated risk factors, indicating the possible relationship between non-arteritic PION and presentation of acute cardioembolic cerebral infarction with severe AF.

## Case report

**Figure 1:**
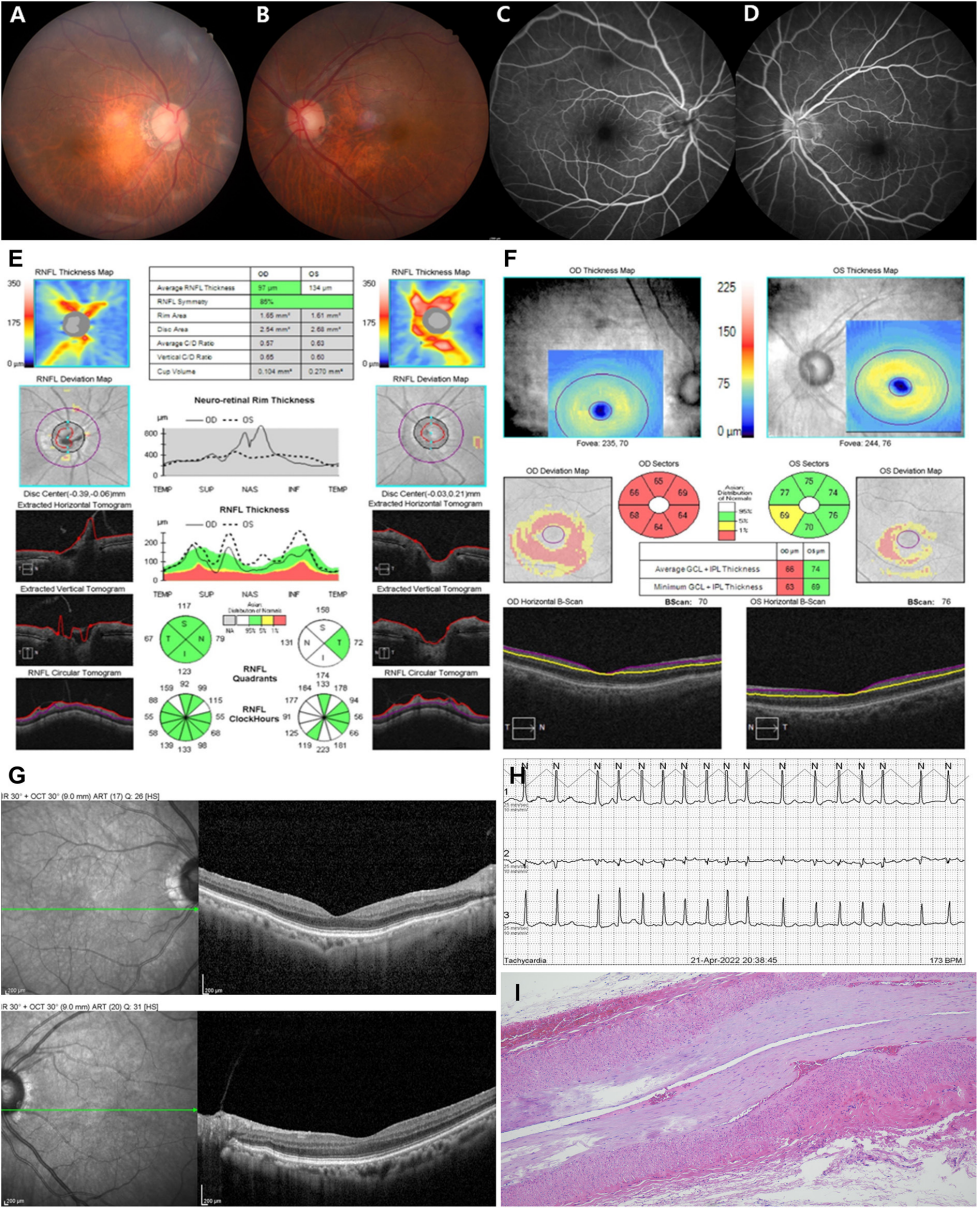
Fundus photography did not show disc swelling in either eye **(A,B)** but mild atrophy in the right eye **(A)**, and fluorescence angiography did not show optic disc leakage or vascular filling defect in both eyes **(C,D)**. Optical coherence tomography (OCT) showed normal average retinal nerve fiber layer (RNFL) thickness in both eyes **(E)** but the thickness of the ganglion cell complex (GCC) layer in the right eye was decreased **(F)**. OCT did not show a significant change in inner retinal layer thickness **(G)**. Twenty-four-hour Holter monitor showed atrial fibrillation (AF) with rapid ventricular response at a rate of 173 beats per minute **(H)**. Photomicrograph of a temporal artery showed mild intimal thickening, otherwise nonspecific **(I)**. ONH, optic nerve head.

An 80-year-old female with hypertension and DM presented to the emergency department with sequential onset bilateral visual loss. One month prior, she developed sudden-onset painless complete visual loss in the right eye followed by the left eye over the next month. The patient did not complain of systemic symptoms such as unexplained weight loss, fever, anorexia, fatigue, scalp tenderness, jaw claudication, or myalgia. She also had no history of transient vision loss, prior trauma, new onset headache, or sleep apnea, but had undergone endovascular coiling due to an aneurysm in the right distal internal carotid artery in 2015. On examination, her visual acuity was light perception in the right eye and finger counting in the left eye. Her pupils were 3 mm in size bilaterally, with relative afferent pupillary defect (RAPD) in the right eye. She had no conjunctival congestion, eyelid swelling, or tenderness over the bilateral temporal region and extraocular movements were normal. Other cranial nerves and sensory motor examinations were normal. Bilateral fundus did not show optic disc edema or hemorrhage in either eye but mild atrophy in the right eye (Figures 1A,B). In addition, fluorescence angiography did not reveal leakages or filling defects but showed mild hypofluorescence in the right eye (Figures 1C,D). Optical coherence tomography (OCT) showed normal average retinal nerve fiber layer (RNFL) thickness in both eyes (Figure 1E) but the thickness of the ganglion cell complex (GCC) layer in the right eye was decreased (Figure 1F). OCT did not show a significant change in inner retinal layer thickness (Figure 1G). Laboratory tests including full blood counts showed normal white blood cells (5.28 × 10^3^/μL; 4.8–10.8 × 10^3^/μL), hemoglobin levels (11.4 g/dL; 12–16 g/dL), and platelet count (256 × 10^3^/μL; 130–450 × 10^3^/μL). Cerebrospinal fluid analysis was non-specific and other measures such as rheumatoid factor, antinuclear antibody, anti-dsDNA IgG, anti-SS A/B, anticardiolipin IgG/IgM, TSH, free T4, anti-aquaporin-4 antibody, myelin oligodendrocyte glycoprotein, oligoclonal bands, and IgG4 were all normal. The C-reactive protein (CRP, 1.41 mg/L; 0–5 mg/L) and erythrocyte sedimentation rate (ESR, 12 mm/h; < 20 mm/h) were also normal at several evaluations throughout the disease course until the 3-month follow-up.

**Figure 2:**
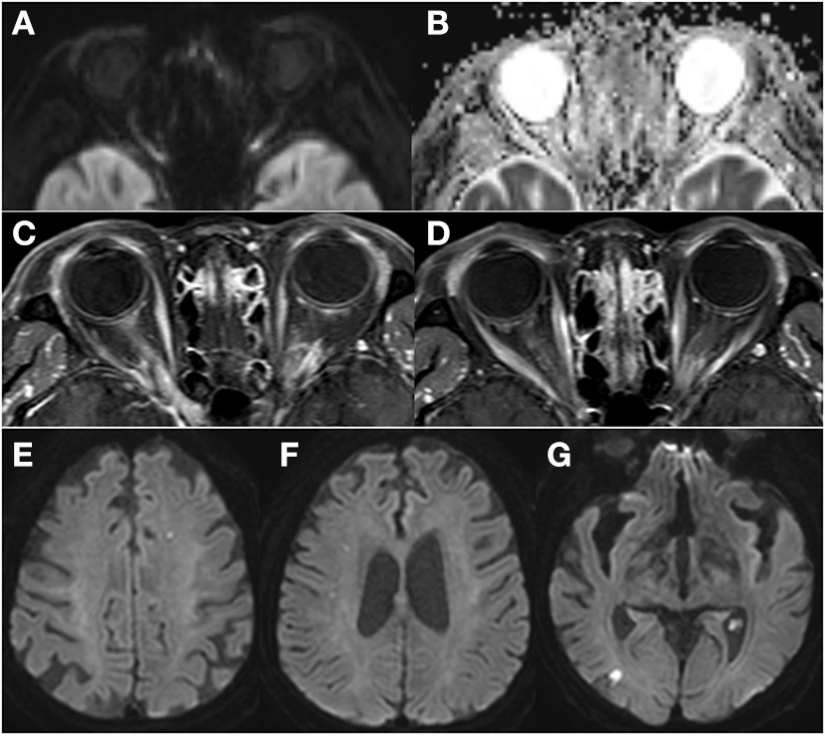
Diffusion-weighted magnetic resonance imaging (MRI) of the brain revealed diffusion restriction in the posterior area of both optic nerves **(A)** which corresponded with the hypo-intensity in the apparent diffusion coefficient (ADC) map **(B)**. Contrast-enhanced axial T1-weighted images revealed enhancement of the bilateral optic nerves in the posterior segment **(C,D)**. Diffusion-weighted MRI of the brain showed multiple scattered lesions of high signal intensity involving bilateral periventricular white matter corresponding to acute embolic stroke **(E–G)**.

**Figure 3:**
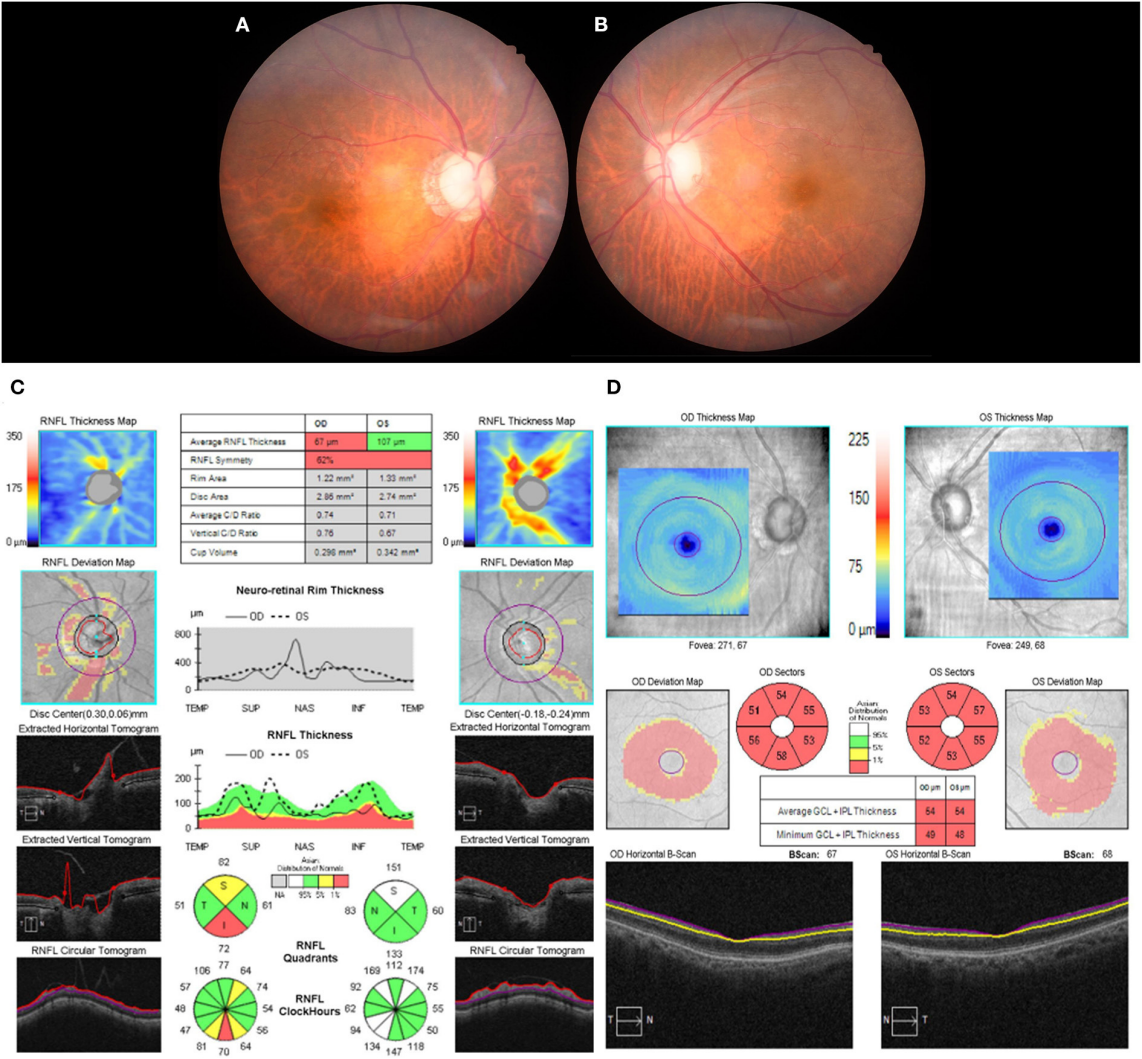
Follow-up fundus photography showed pale and atrophic disc in both eye **(A,B)**, and cptical coherence tomography (OCT) retinal nerve fiber layer (RNFL) analysis showed normal RNFL thickness in the left eye but RNFL thinning in the right eye **(C)**. The thickness of the ganglion cell complex (GCC) demonstrated severe thinning in both eyes **(D)**.

Electrocardiogram (ECG) showed AF with rapid ventricular response, and the 24-h Holter monitor showed AF with rapid ventricular response at a rate of 173 beats per min (Figure 1H). Systolic and diastolic blood pressure were measured every 3 h during hospitalization, including at night, and ranged from 129/69 mmHg−157/93 mmHg while the patient was on antihypertensive medication. Two-dimensional echocardiography revealed severely enlarged left atrium with normal ventricular systolic function. Diffusion-weighted magnetic resonance imaging (DWI) of the brain showed diffusion restriction in multiple scattered areas involving bilateral periventricular white matter corresponding to acute embolic stroke (Figures 2E–G) and in the posterior area of both optic nerves (Figure 2A), which also corresponded with the hypointensity in the apparent diffusion coefficient (ADC) map (Figure 2B). Contrast-enhanced axial T1-weighted images revealed enhancement of optic nerves (Figures 2C,D). Heterogeneous echogenic plaques in the left internal carotid artery with mild stenosis on carotid Doppler ultrasonography and arteriosclerotic changes in brain MRA examination were observed. For diagnosis, segmental resection of the left temporal artery was performed. Histologically, the temporal artery showed intimal thickening but no evidence of arteritis (Figure 1I). The patient was treated with 500 mg intravenous methylprednisolone for 5 days and an anticoagulant (apixaban) with an antiplatelet agent (cilostazol). She was discharged on hospital day 18 with no change in visual status. At 2-month follow-up, her visual acuity was confirmed as no light perception in both eyes. Bilateral fundus showed pale and mild atrophic optic disks (Figures 3A,B). Follow-up OCT showed normal average RNFL thickness in left eye but RNFL thinning in the right eye (Figure 3C), and the thickness of the GCC layer was decreased in both eyes (Figure 3D).

## Discussion

Herein, we describe a patient who presented with bilateral sequential visual loss associated with multiple embolic infarctions and severe AF. Non-arteritic PION is exceedingly rare compared with non-arteritic AION (3, 4), and the actual epidemiology and pathomechanism remain unclear. In contrast to the anterior portion of the optic nerve, which is supplied by both the pial plexus and the central retinal artery, the posterior portion of the optic nerve is only supplied by the circumferential pial capillary plexus derived from distal collateral branches of the ophthalmic artery that surrounds it (8). In particular, the core section of the posterior optic nerve is poorly vascularized with just a few penetrating capillaries capable of autoregulatory control, causing this area to be at greater risk of ischemia (8).

Although only a few population-controlled studies on ION and its risks have been performed, and the pathophysiologic role of AF is poorly understood, ischemia in the posterior portion of the intraorbital optic nerve could result from microembolism of the perineural pial plexus, the principal source of blood supply to this segment. In a previous study, the prevalence of AF in subjects with retinal vascular occlusive disease and ION was analyzed and was approximately 16.0–18.4% in retinal arterial occlusion (RAO) or retinal venous occlusion (RVO), and 14.8% in ION with no significant differences between groups including ischemic stroke (26%) (9). In another case report, paroxysmal AF was causally associated with RAO in a patient without other risk factors for RAO (10). In most cases, detecting paroxysmal AF is challenging because episodes are often brief, unpredictable, and frequently asymptomatic or accompanied by unspecific ION symptoms. Although ION is a pathogenetically different clinical entity from cerebral ischemic stroke, systemic risk factors for stroke and ION are similar. Furthermore, although systematic analysis of a larger patient cohort for establishing the role of AF in ION has not been performed, especially in PION, the concurrent cerebrovascular events and PION in the current case are likely associated with a micro-embolism mostly due to uncontrolled AF. The thromboembolic risk in paroxysmal and persistent AF, and even short episodes lasting a few minutes, are associated with higher rates of ischemic events (11, 12). In addition, the prevalence of AF observed was similar in ION and ischemic stroke patients, and history of a previous stroke was more common in the ION group (9). Furthermore, an elevated stroke risk after non-arteritic AION has been recently reported (13). In several retrospective studies, significant increases in the risk of cardiovascular events or death following non-arteritic AION were reported; however, an increase in cardiovascular or cerebrovascular events following non-arteritic AION was not observed in other published studies (14–16).

The degree or temporal course to which eyes are affected varies significantly and bilateral involvement can occur (3). In a series by Sadda et al., PION was bilateral in 21% of non-arteritic PION, 50% of arteritic PION, and 54% of perioperative PION patients (3); in Hayreh's series, the corresponding incidence was 25, 17, and 75% respectively (4). Reported cases of bilaterally involved PION in the literature have variable etiologies, however, the occurrence of bilateral PION concurrent with multifocal embolic infarction and uncontrolled AF without any hemodynamic instability has not been reported to date (Table 1). In the present case study, the main differential diagnosis for acute bilateral presentation of optic neuropathy was bilateral retrobulbar optic neuritis or bilateral arteritic or non-arteritic PION. In addition, the enlarged cupping in the right eye, the presentation of bilateral severe vision loss in an elderly female, and the enhanced orbital MRI may suggest a previous optic neuropathy of arteritic (GCA) form in the right eye. However, the patient did not complain of any systemic symptoms such as unexplained weight loss, fever, fatigue, scalp tenderness, jaw claudication, or myalgia. In addition, laboratory studies showed normal blood counts including a platelet count and the CRP and ESR were normal at several evaluations throughout the disease course until the 2-month follow-up. She also showed a negative temporal artery biopsy and diffusion restriction was seen on DWI in the posterior area of both optic nerves with corresponding reduced ADC values. Meanwhile, the optic nerve cupping in the right eye could be evidence of pre-occurred central retinal artery occlusion (CRAO) from AF-induced embolus which would no longer be present. However, OCT did not show a significant change in inner retinal layer thickness or inner retinal hyperreflectivity which could be a biomarker for CRAO, in the current patient. Therefore, the presence of painless and maximal visual loss at the onset with no improvement, older age with DM, and uncontrolled AF, favored a diagnosis of non-arteritic PION for visual obscuration in the current patient. Furthermore, stable clinical status without aggravation of visual impairment or systemic symptoms even after stopping prednisone during the 3-month follow-ups diagnostically supported non-arteritic PION. In addition, MRI showing hyperintense signals of bilateral optic nerve on DWI with contrast enhancement of the optic nerve can be used to diagnose IONs (28–30). Several GCA diagnostic criteria proposed by the American College of Rheumatology also did not support a diagnosis of GCA for our patient (31, 32). PION should also be considered in patients presenting with sudden painless bilateral vision loss with normal fundoscopy in the background of systemic disease such as DM or cardiac disease such as AF.

**Table 1:** Characteristics of reported bilateral sequential PION cases.

**Age/sex**	**Type of PION**	**Risk factor**	**Initial visual acuity**	**Treatment**	**Prognosis**
67/M (17)	Postoperative	Bilateral radical neck dissection, hypotension	NLP both	Dexamethasone + transfusion	NLP both (died)
68/F (18)	Postoperative	Lumbar spine surgery (Blood loss: 3,000 cc), DM	NLP both	Transfusion	NLP both
33/F (19)	Postoperative	Lumbar spine surgery (Blood loss: 1,200 cc)	HM both	Hydration + transfusion	20/25 both
42/M (20)	Non-arteritic	HTN, chronic renal insufficiency, hemodialysis	NLP both	Intravenous corticosteroid therapy for 36 h	NLP both
76/M (21)	Non-arteritic	Use of sildenafil, HTN, hyperlipidemia	NLP both	-	CF right; HM left
35/F (22)	Non-arteritic	Type 1 DM, DKA	NLP both	Hydration + insulin treatment	NLP both
48/F (23)	Postoperative	Cardiac transplantation	NLP both	Hydration + transfusion	NLP both
57/F (24)	Postoperative	Spine surgery	NLP both	Transfusion	NLP, LP
39/F (25)	Non-arteritic	Use of sildenafil for pulmonary hypertension	CF at 3 feet both	-	CF at 3 feet both
80/M (26)	Arteritic	GCA	NLP both	Methylprednisolone 1 g for 5 days	NLP both
45/F (27)	Non-arteritic	DM (HbA1c 12.1)	CF right; LP left	Insulin therapy + methylprednisolone 1 g for 5 days	6/60 right; LP left
80/F^*^	Non-arteritic	AF, atherosclerosis, DM, HTN	LP right; CF right	Methylprednisolone 1 g for 5 days + anticoagulant + antiplatelet agent	LP right; CF right

PION, posterior ischemic optic neuropathy; M, male; F, female; NLP, no light perception; LP, light perception; CF, count fingers; HM, hand motion; OD, right eye; OS, left eye; OU, both eyes; GCA, giant cell arteritis; AF, atrial fibrillation; DM, diabetes mellitus; DKA, diabetic ketoacidosis; HTN, hypertension.

* our case.

The visual prognosis for patients with non-arteritic PION is usually poor. Although in a randomized non-arteritic PION study, patients who received systemic corticosteroid treatment showed improved visual acuity compared with subjects without corticosteroid treatment (33), the current patient treated with high-dose steroid therapy did not show a change in vision. To the best of our knowledge, the present case is the first in which the possible relationship between non-arteritic PION and uncontrolled AF and embolic cerebral infarction was suggested. In conclusion, the possible cause and pathophysiology remain unclear; however, hypoperfusion of the optic nerve circulation associated with embolic lesions should be suspected, and early detection is essential in the management of ION.

## Data availability statement

The raw data supporting the conclusions of this article will be made available by the authors, without undue reservation.

## Ethics statement

This study was reviewed and approved by the Institutional Review Board at Jeonbuk National University Hospital. The patients/participants provided their written informed consent to participate in this study. Written informed consent was obtained from the individual(s) for the publication of any potentially identifiable images or data included in this article.

## Author contributions

E-SL, H-JL, SH, J-JK, and M-JC wrote sections of the manuscript. S-YO supervised and contributed to the study concept and revised the manuscript. All authors read and approved the submitted version.

## Funding

This work was supported by a National Research Foundation of Korea (NRF) grant funded by the Korean government (Ministry of Science and ICT) (No. 2022R1A2B5B01001933) and by the Biomedical Research Institute Fund, Jeonbuk National University Hospital.

## Conflict of interest

The authors declare that the research was conducted in the absence of any commercial or financial relationships that could be construed as a potential conflict of interest.

## Publisher's note

All claims expressed in this article are solely those of the authors and do not necessarily represent those of their affiliated organizations, or those of the publisher, the editors and the reviewers. Any product that may be evaluated in this article, or claim that may be made by its manufacturer, is not guaranteed or endorsed by the publisher.
